# Beneficial effects of linoleic acid on cardiometabolic health: an update

**DOI:** 10.1186/s12944-024-02246-2

**Published:** 2024-09-12

**Authors:** Kristina H. Jackson, William S. Harris, Martha A. Belury, Penny M. Kris-Etherton, Philip C. Calder

**Affiliations:** 1 OmegaQuant Analytics, 5009 W. 12th St, Suite 8, Sioux Falls, Sioux Falls, SD 57106 USA; 2 Fatty Acid Research Institute, Sioux Falls, SD USA; 3 Sanford School of Medicine, University of South Dakota, Sioux Falls, SD USA; 4 Department of Food Science and Technology, The Ohio State University, Columbus, OH USA; 5 Department of Nutritional Sciences, The Pennsylvania State University, University Park, PA USA; 6 School of Human Development and Health, Faculty of Medicine, University of Southampton, Southampton, UK; 7 NIHR Southampton Biomedical Research Centre, University Hospital Southampton NHS Foundation Trust and University of Southampton, Southampton, UK

**Keywords:** Linoleic acid, Alpha-linolenic acid, Cardiovascular disease, Type-2 diabetes, Cardiometabolic health, Omega-6 fatty acids

## Abstract

Linoleic acid (LA), as a part of the wider debate about saturated, omega-6 and omega-3 fatty acids (FAs) and health, continues to be at the center of controversy in the world of fatty acid research. A robust evidence base, however, demonstrates that higher intakes and blood levels of LA are associated with improved cardiometabolic health outcomes. LA lowers total and low-density lipoprotein cholesterol when compared with saturated fatty acids and carbohydrates. Using large prospective datasets, higher blood levels of LA were associated with lower risk of coronary heart disease, stroke and incident type-2 diabetes mellitus compared with lower levels, suggesting that, across the range of typical dietary intakes, higher LA is beneficial. Recent trials of LA-rich oils report favorable outcomes in people with common lipid disorders. However, an LA intake that is too high can impair endogenous synthesis of eicosapentaenoic acid (EPA) from alpha-linolenic acid (ALA), but the threshold at which this becomes clinically relevant is not known. In the absence of a significant intake of EPA and docosahexaenoic acid, an ideal dietary ratio of LA and ALA may be theoretically useful as it provides insight into the likely extent of endogenous EPA synthesis from ALA. Updating dietary reference intakes (DRIs) for LA and ALA is needed; however, there are insufficient data to establish RDAs for these fatty acids. The omega-6 (n-6) to omega-3 (n-3) PUFA ratio is not informative and does not shed meaningful insight about the amount of individual fatty acids in each class needed to confer health benefits.

## Introduction

In the nutrition research community focusing on fatty acids (FAs) and health, there is significant controversy about what the optimal intake of linoleic acid (LA) ought to be. Virtually everyone agrees that an intake of LA resulting in skin lesions is too low, but there is debate about the health impact of the current intake of LA in the US (around 12–17 g/d or about 6% of energy) provided largely by seed oils or foods that contain them. There is no question that LA has increased in the US food supply over the past century [1], but whether this is good or bad, the root cause of many of our modern ailments or a contributor to the general improvement in health observed over the same time period, is controversial. Driven largely by the need to remove *trans* fats from the food supply, seed oil production companies have been changing the FA composition of edible oils by selective breeding to convert high LA oils (like grapeseed, cottonseed, sunflower, canola and corn) into “high-oleic” versions. This change lowers the amount of polyunsaturated FAs (PUFAs), specifically LA and alpha-linolenic acid (ALA), and increases the amount of monounsaturated FAs (MUFAs) in these oils and thus in the US food supply. Whether this change is healthful or harmful for the public is not known. Here, we outline the recent evidence supporting the hypothesis that higher intakes of LA are associated with improvements in relevant biomarkers and with lower risk for developing cardiometabolic diseases, and we address some of the most common concerns about higher LA intakes and levels.

## Linoleic acid: status, usual intakes, and changes in food sources in the US food supply

LA is an essential nutrient since it cannot be synthesized in the body. It is an omega-6 (n-6) PUFA with 18 carbons and 2 double bonds. Severe deficiency of LA results in dermal conditions, such as rough, dry and scaly skin [2]. Traditional dietary oils that are good sources of LA include the seed oils noted above, with the high-oleic versions of sunflower, soybean and safflower providing some LA, but less than the native oils. These plant sources of LA have been used in the food industry in their native form, and following partial hydrogenation, which can result in the generation of *trans* FAs. Although high LA oils are the food source with the highest proportion of LA per serving, the most common dietary sources of LA in the US diet are mixed grain, meat, potato and seafood dishes containing these oils [3].

High LA seed oils are a novelty in the human diet, being introduced in the 1800s with the surplus of cottonseeds from the US cotton industry. Cottonseed oil is microbiologically stable and was originally produced for industrial applications such as lubricants. Further development led to the production of cottonseed oil suitable for human consumption containing about 50% LA and less than 0.4% ALA. Eventually, this was partially hydrogenated to produce shortenings (e.g., Crisco^®^) which was introduced in 1911 [4]. Such products were particularly high in *trans* FAs which, in the late 20th century, were recognized as harmful and have been largely banned as food ingredients [5, 6].

Although high LA oils came to be equated with “high PUFAs”, these oils overwhelmingly have only one PUFA (i.e., LA). By the 1950s, high PUFA (i.e. LA) sources were regarded as healthful. In 1957, the American Heart Association (AHA) issued a report that summarized the evidence about the relationship between diet and atherosclerosis and concluded that diet likely played an important role, and that the balance between saturated and unsaturated fat may also be important [7]. In 1961, an ad hoc AHA committee updated the earlier report and concluded that diet should be modified by decreasing total fat, saturated fatty acids (SFAs) and cholesterol and increasing PUFAs [8]. Interestingly, in 1968 it was recommended that PUFAs “should probably comprise twice the quantity of SFA”. The history of the AHA dietary fat recommendations from 1957 to 2015 has been published [9].

### Intake of LA

In North America, Adequate Intake (AI) levels for LA have been set reflecting their approximate daily intakes as there was not enough evidence to establish an estimated average requirement (EAR) or a recommended dietary allowance (RDA) [10]. Currently, AIs for LA for adults are 12 g/d for women and 17 g/d for men, which generally equates to ∼ 6% of daily energy intake [11, 12]. The Acceptable Macronutrient Distribution Range for n-6 PUFA (linoleic acid) is 5 to 10% of energy [13]. The AHA also recommends an LA intake of 5–10% of energy [14]. On a 2,000 kcal/d diet, 5–10% of energy intake (i.e. 100–200 calories) equates to 11–22 g/d of LA. At the other end of the intake spectrum, severe essential FA deficiency symptoms are avoided with as little as 2% of energy intake (or ∼ 4.5 g/d), but even this may be as much as 10-times higher than actual needs [15]. Here, then is the issue: is 5 g/d (or maybe even 1 g/d) or 10–20 g/d of LA best for “health?” Obviously, defining health is important in this discussion, but here we mainly focus on risk of cardiovascular disease (CVD) and type-2 diabetes mellitus (T2DM), together with cardiometabolic disease (CMD). In contrast, severe deficiency would be characterized by a breakdown of skin and epithelial barriers leading to dermatitis, dehydration and increased risk of infection.

Mathematical modelling has been used to investigate the extent to which the replacement of traditional seed oils with high-oleic acid oils might impact LA intakes: replacing 50% or more of traditional soybean and canola oils in foods with high-oleic alternatives could clearly reduce intake of LA and ALA in adults [16] and in children [17]. Specifically, such a replacement would theoretically reduce current LA intakes of 20 g/d to 14 g/d [3]. Whether these changes could inadvertently increase the prevalence of CMDs is not known, but this question could be explored through modelling exercises and clinical trials investigating the impact on risk factors of high oleic oils vs. traditional oils rich in LA.

### Blood levels of LA

Dietary LA is overwhelmingly the greatest contributor to the body’s pool of n-6 PUFAs followed by dietary arachidonic acid (ARA). ARA blood levels are primarily affected by ARA intake, but endogenous metabolism and intake of EPA and DHA are also known to have an effect [18]. Other n-6 PUFAs in the blood are largely derived from metabolism and include gamma linolenic, di-homo-gamma linolenic, adrenic and docosapentaenoic acids. While both metabolism and dietary intake play a role in blood FA levels, circulating LA is largely driven by intake, as demonstrated elsewhere [19, 20]. Typical US levels of LA in various blood pools are shown in Table 1.

**Table 1:** Approximate range of LA and ARA (as a percent of total FAs) in serum, red blood cells and whole blood in US adults

Sample Type	*N*	LA	ARA	Reference
Serum (%)	2,261	34–35%	6–7%	Murphy et al. [21]
Red blood cells (%)	155,586	11–13%	15–16%	Schuchardt et al. [22]
Whole blood (%)	11,315	21–25%	8–10%	Marklund (pending)

## Recent epidemiological findings on blood n-6 fatty acid levels and incident cardiometabolic disease

The studies discussed below are from a group of nutrition research centers called the Fatty Acids and Outcomes Research Consortium (FORCE) [23]. FORCE performs meta-analyses of *de novo*, cohort-level data on the relationships between blood or tissue levels of a given FA or FA group and various disease outcomes. This approach is more powerful and less subject to publication bias than traditional meta-analyses that only use data from published studies, which can vary by inclusion/exclusion criteria, covariates, and exposure and outcome definitions. In FORCE research projects, all cohorts perform their individual analyses using harmonized exposure and outcome definitions, uniform covariate lists, and systematic subgroup analyses. Moreover, multiple sample types, e.g. RBCs, plasma, plasma phospholipids, are harmonized using inter-quintile range analyses (IQ_5_R; i.e., comparing approximately 90th vs. 10th percentile). As such, the following studies will reference “blood levels” as the results are consistent among multiple sample types. FORCE has published two studies examining the relationships between n-6 PUFA levels and risk for future CMD [24, 25].

The relationships between coronary heart disease (CHD) risk and blood n-6 PUFAs were examined by Marklund et al. [25]. This study included 19 prospective cohorts from 16 countries with a total of 45,637 individuals followed for a median of 10 years. In a meta-analysis of the multivariable adjusted findings from each cohort, the percent difference in risk for several CVD outcomes per IQ_5_R of in vivo LA and AA levels is shown in Table 2.

**Table 2:** Summary of FORCE findings for the relative risk reductions (95% CI) per interquintile range (IQ_5_R; i.e., comparing the 90th vs. the 10th percentile) of both blood LA and ARA for coronary heart disease (CHD) [25], cardiovascular disease (CVD) [25] and type 2 diabetes mellitus (T2DM) [24]. Values in bold were statistically significant, *p* < 0.05

Outcome	*N*-6 PUFAs	
	LA	ARA
CHD (total)	-6% (-12 to 0)	-1% (-6 to 4)
CVD		
Total	**-7% (-12 to -1)**	-5% (-10 to 1)
Fatal	**-22% (-30 to -15)**	-6% (-14 to 2)
Ischemic Stroke	**-12% (-21 to -2)**	-1% (-10 to 10)
T2DM	**-35% (-40 to -18)**	-4% (-12 to 5)

Risk for total CVD events was inversely associated with LA (7% lower risk per IQ_5_R), but ARA levels were unrelated to risk (Table 2). Consistent with this biomarker-based study was the meta-analysis of dietary intake-based studies by Farvid et al. [26] which included 13 prospective cohort studies, over 310,000 individuals among which were nearly 13,000 CHD events and 5,900 CHD deaths. They found that the highest (vs. the lowest) intake categories of LA were associated with a 15% lower risk of CHD events and a 21% lower risk of CHD deaths. Another FORCE publication from Wu et al. [24] reported the associations between n-6 PUFA biomarkers and risk for incident T2DM. This study included 39,740 participants in 20 prospective cohort studies across 10 countries, with a median follow-up time of about 9 years. Risk for incident T2DM was 35% lower per IQ_5_R for LA but was unrelated to ARA levels (Table 2).

Thus, risk for these major clinical outcomes was significantly lower with higher in vivo levels of LA. Although all associations with ARA levels were non-significant, relative risk point estimates in all cases were < 1, suggesting at the very least, that ARA was not linked with increased risk for any of these outcomes. Wang recently surveyed the epidemiologic data for LA and CVD and concluded, “abundant evidence from prospective cohort studies and randomized controlled trials (RCTs) have shown that high n-6 PUFA intake plays an important role in the dietary prevention of CVD” [27]. A recent paper from the UK Biobank examining plasma PUFA levels as predictors of total and cause-specific mortality stated that, “…[O]mega-3 and omega-6 PUFAs in plasma were consistently and inversely associated with all-cause, cancer, and CVD mortality, with omega-3 showing stronger effects” [28]. Taken together, these epidemiologic findings strongly suggest that higher LA (and in some settings, ARA) levels are linked with improved health outcomes.

## LA improves lipid and glycemic status

Many adults in the US and other countries are affected by the major CMDs [29]. There is a strong pathological relationship between CV conditions and those involving insulin resistance. Individuals with a genetic propensity for hyperlipidemia are at elevated risk for obesity and T2DM [30]. The prevalence of lipid disorders among adults in the US is 33.9% [29]. A meta-analysis of 60 RCTs by Mensink et al. demonstrated that when replacing carbohydrates with different kinds of fats, the total: high density lipoprotein (HDL)-cholesterol ratio was the most improved with PUFAs compared to replacement with SFAs or MUFAs [31]. Vessby et al. conducted a clinical trial in adults with the three most common dyslipidemias (high cholesterol, high triglycerides, and the combination) substituting a diet enriched in LA (vs. usual diets) for only two weeks significantly improved fasting levels of very low density lipoprotein (VLDL)-, low density lipoprotein (LDL)- and HDL-cholesterol [32]. In addition, glucose intolerance was significantly improved in adults with hypertriglyceridemia [32]. Findings from this study evaluating risk factors for both CVD and T2DM support a causal relationship between dietary LA and reduced risk for CMDs.

Blood or tissue levels of LA have been shown to be positively associated with insulin sensitivity [32–39] and with reduced risk for developing insulin resistance as well as T2DM by several researchers [38, 40–44]. RCTs have shown that adding oils rich in LA improves glycemic control [45, 46], improves insulin sensitivity [47], and reduces central obesity [46–48] when compared to a mixed fat diet. The consistent findings across the observational studies which are supported by RCT data strongly suggest that the association between LA and reduced risk for CVD and T2DM is not simply coincidental but causal.

The mechanisms by which LA reduces the likelihood of developing insulin resistance are not well understood. Reducing dyslipidemia is likely involved but probably not the full explanation. It is possible that LA and LA-derived oxylipin metabolites alter glucose/insulin metabolism through activating peroxisome proliferator-activated receptors (PPARs) [49–52]. For example, supplementing the diet of post-menopausal women with metabolic syndrome (but not T2DM) with LA (approx. 6.9 g/day on top of habitual intake from the diet) for 16 weeks increased plasma LA and LA-derived oxylipins and levels of the insulin-sensitizing adipokine, adiponectin [53], the gene for which is PPAR-responsive [54].

More research is needed to address whether LA *causes* (versus, is related with) lowered risk for CVD and T2DM in study populations with greater diversity and in the context of other background diets.

## Randomized controlled trials of LA and cardiovascular disease

A 2011 meta-analysis of seven RCTs in which PUFA-rich vegetable oils replaced SFAs and the effects on coronary heart disease were examined found a 19% overall reduction in risk [55]. This finding is generally consistent with epidemiological observations. However, Ramsden et al. in a series of papers raised questions about the validity and interpretation of several of these studies, suggesting that in fact, higher LA intakes might at best be neutral, and possibly even adverse [56, 57]. In response to these reports, a variety of rebuttals were published that challenged the Ramsden conclusions and re-affirmed the beneficial effects of PUFAs substituting for SFAs [58–60]. A detailed review of this particular controversy is, however, beyond the scope of the present paper.

## Addressing other concerns about higher LA intakes

There is a long-held assumption that higher LA in the diet worsens inflammation because higher LA was assumed to lead to higher tissue ARA levels, and ARA can be converted into some pro-inflammatory mediators [61]. However, this has been refuted in several studies [62–65]. We are unaware of any RCTs showing that supplementing the diet with LA-rich oils increases markers of inflammation or dysregulated metabolism [66, 67]. In fact, higher levels or dietary fortification of LA reduced markers of inflammation in several studies [45, 46].

Another argument for LA being harmful at current intakes is that it may reduce endogenous EPA (and less so DHA) production from ALA, the essential omega-3 (n-3) PUFA. A review of the health effects of EPA and DHA is beyond the scope of this review, but these have been recently summarized [68–71]. It is clear that LA and ALA compete at the substrate level for the endogenous production of longer-chain n-6 and n-3 PUFAs as first elucidated in rat feeding studies in the 1960s [72]. Metabolic suppression of tissue accretion of n-3 PUFAs by high dietary LA was established by the mid-1970s, suggesting that one potentially adverse impact of high LA intake would be reduced tissue n-3 PUFA levels.

Studies manipulating the intake of LA while keeping ALA intakes constant reported effects on n-3 PUFA status. For example, Ramsden et al. fed 52 volunteers with a low LA diet for 12 weeks [73] and measured the changes in erythrocyte EPA + DHA, LA and ARA levels. Decreasing LA intake from 7.4% of energy intake to 2.4% had the following effects on erythrocyte PUFAs: EPA + DHA increased from 3.7 to 4.1%; LA decreased from 12.2 to 10.5%, and ARA decreased from 14.2 to 13.1%. Liou et al. [74] compared a diet providing ∼ 4% energy as LA with a diet providing ∼ 11% energy, both containing ∼ 1% of energy as ALA. The higher LA diet decreased plasma phospholipid EPA from 1 to 0.5% of total FAs [75], a large percentage decrease but a minimal absolute reduction. These observations suggest that the dietary ratio of LA to ALA could be a key determinant of endogenous EPA synthesis (in the absence of preformed EPA in the diet), although its effects on DHA levels are less clear. Thus, a deleterious consequence of a too high intake of LA, within an all-too-common diet that is low in seafood, is constrained EPA synthesis, resulting in lower EPA status and possible loss of some of the physiological and health benefits of EPA. However, caution is needed even when considering the LA to ALA ratio. Goyens et al. [76] compared the effects of two diets both with an LA to ALA ratio of 7. One diet contained 3% of energy as LA and 0.4% as ALA and the other contained 7% of energy as LA and 1.1% as ALA. Plasma phospholipid EPA increased by over 50% on the lower PUFA diet and by only 25% on the higher PUFA diet. Thus, a quantitatively different outcome was achieved by using two different diets with the same LA to ALA ratio. The authors argued that absolute amounts of these PUFAs are more important than their ratio.

It is important to note that the elevations in EPA reported in trials reducing intakes of LA are typically much smaller than seen with increased intake of preformed EPA, and as noted, this strategy does not increase DHA status [74]. Thus, whether the changes in EPA accompanying a low LA diet would have net favorable or adverse effects on overall health is unclear and remains an untested hypothesis. It is possible that whatever potential adverse effects might arise from somewhat lower EPA levels could, at least in theory, be offset by the benefits of increased LA-derived mediators and other effects of LA on cell function. Clearly, further research is needed to sort out these questions.

## Is there any utility to the n-6 to n-3 PUFA ratio?

The ratio of n-6 to n-3 PUFAs is widely used in both professional and lay literature. Nevertheless, its validity and utility has been questioned [77, 78], and there are no official recommendations for an optimal ratio in either the diet or blood, to our knowledge [79]. Assumptions underlying the use of this ratio include (1) that all n-6 PUFAs are “bad”, that all n-3 PUFAs are “good” and that all n-3 PUFAs oppose the action of all n-6 PUFAs; (2) that all n-6 PUFAs are functionally equivalent, as are all n-3 PUFAs; (3) that all ways of changing the ratio are equivalent, and (4) that higher LA intakes lead to higher ARA levels. None of these assumptions is correct, as we describe below.

On the first assumption, benefits of higher dietary/tissue LA levels have already been discussed. Second, although many oxylipins produced from ARA can be viewed as undesirable in some contexts [80], ARA also gives rise to lipoxins which are beneficial specialized pro-resolving mediators [81], just like resolvins, protectins and maresins produced from n-3 PUFAs [82]. LA, gamma-linolenic acid, dihomo-gamma-linolenic acid and ARA (all n-6 PUFAs) are not functionally equivalent; just as ALA, stearidonic acid, EPA, docosapentaenoic acid (DPAn-3) and DHA (all n-3 PUFAs) are not functionally equivalent. Therefore, each of these PUFAs should not be given the same “weighting” in the numerator and the denominator of the ratio. On the third assumption, the fact that not all ways of changing the ratio are equivalent is well illustrated by considering the changes in plasma EPA% reported in the study of Goyens et al. [76] described earlier with higher vs. lower total PUFA diets with the same LA: ALA ratio. This is further illustrated by a study in which subjects were randomized to ALA (6.6 g/d) or EPA + DHA (3.6 g/d) for 8 weeks [83]. The n-6 to n-3 PUFA ratio of the diets was 2.1 and 2.9, respectively. The RBC EPA + DHA actually decreased by -0.13% points in the ALA group but increased by 6.7 points in the EPA + DHA group. Thus, similar n-6 to n-3 PUFA ratios had very different effects on a standard marker of tissue n-3 PUFA status. Thies et al. conducted a similar experiment in older adults with similar results [84]. These findings indicate that the intake of individual PUFAs is what matters, and that an n-6 to n-3 PUFA ratio can be a distraction. Finally, in human studies, higher LA intakes have no effect on ARA levels [65, 85, 86].

Additionally, the utility of the n-6 to n-3 PUFA ratios is limited because n-6 to n-3 PUFA ratios can be constructed for the diet, as well as for blood (and plasma, and RBCs, and plasma phospholipids and indeed, every lipid pool) and each will be different. Setting a target n-6 to n-3 PUFA ratio would have to be specific to the lipid pool of interest, which is clinically unwieldy.

Theoretically, it seems that in the absence of significant intake of preformed EPA and DHA, a dietary LA to ALA ratio could be useful. However, the previously described studies have demonstrated the limitations of using the ratio in practice, such that it is very likely that the absolute intakes of LA and ALA are more important than their ratio. When EPA and DHA are consumed in reasonable amounts, neither the LA to ALA nor the n-6 to n-3 PUFA ratio of the diet is useful [77, 87]. All in all, the use of the n-6 to n-3 PUFA ratio, dietary or otherwise, should be discontinued.

## Summary

The health impact and underlying mechanisms of action of PUFAs have been studied for several decades now. Based on current knowledge there are recommendations for intake of LA, ALA and EPA and DHA, but they are based largely on population averages and not on optimal intakes, which are yet to be determined. LA and ALA are the essential FAs and are precursors of longer chain, more unsaturated PUFAs and a wide array of oxylipins. LA has important roles in skin and in regulating cholesterol homeostasis. Dietary intakes of LA have increased over the years with the introduction of LA-rich oils and margarines and through grain-feeding to livestock, but the extent to which this has been for good or for ill remains controversial. A high intake of LA does somewhat diminish endogenous biosynthesis of EPA from ALA, but how this affects overall risk for chronic disease is unknown as is the definition of “high intake.” These should be the focus of future research. Recent publications using large datasets report that higher blood levels of LA are associated with lower risk of CHD, stroke and T2DM compared with lower levels, confirming that across the range of normal dietary intakes, higher LA is beneficial to health. Recent trials of LA-rich oils report favorable outcomes on total and LDL cholesterol, glucose homeostasis and insulin sensitivity in individuals with common lipid disorders. Current intakes tend to fall within the recommendations for LA intake (5–10% of energy) that are associated with improved cardiometabolic health, but more research is needed to determine the cut-points for LA intake or blood levels that would indicate the need for an individual to increase or decrease LA intake. In summary, there is now good evidence that LA has cardiometabolic health related benefits, and individuals with lower intakes of LA would be expected to benefit from increasing LA intake.

## Data Availability

No datasets were generated or analysed during the current study.

## Abbreviations

AHA: American Heart Association
AI: Adequate intake
ALA: Alpha linolenic acid
ARA: Arachidonic acid
CMD: Cardiometabolic disease
CV: Cardiovascular
CVD: Cardiovascular disease
DHA: Docosahexaenoic acid
DPAn-3: Omega-3 docosapentaenoic acid
DRI: Dietary reference intake
EAR: Estimated average requirements
EPA: Eicosapentaenoic acid
FA: Fatty acids
FORCE: Fatty Acids and Outcomes Research Consortium
HDL: High-density lipoprotein
IQ_5_R: Inter-quintile range
LA: Linoleic acid
LDL: Low-density lipoprotein
MUFA: Monounsaturated fatty acids
n-6: Omega-6
n-3: Omega-3
PPARs: Proliferator-activated receptors
PUFA: Polyunsaturated fatty acid
RCTs: Randomized controlled trials
RDA: Recommended dietary allowance
SFA: Saturated fatty acid
T2DM: Type-2 diabetes mellitus
VLDL: Very low-density lipoprotein
